# Authors’ reply: “Chronic high consumption of energy drinks and cardiovascular risk in adolescents—results of the EDKAR-study”

**DOI:** 10.1007/s10654-025-01343-5

**Published:** 2026-02-21

**Authors:** Juliane Menzel, Fabian Spinka, Maria J. Pie, Andrea Deichl, Sven Knüppel, Anke Ehlers, Britta Nagl, Frank Edelmann, Cornelia Weikert

**Affiliations:** 1 Department Food and Feed Safety in the Food Chain, German Federal Institute for Risk Assessment, Max-Dohrn-Straße 8-10, 10589 Berlin, Germany; 2 Institute of Social Medicine, Epidemiology and Health Economics, Charité – Universitätsmedizin Berlin, Corporate Member of Freie Universität Berlin and Humboldt-Universität zu Berlin, Berlin, Germany; 3 German Center for Cardiovascular Research (DZHK), Partner Site Berlin, Berlin, Germany; 4 Department of Cardiology, Angiology and Intensive Care Medicine, Deutsches Herzzentrum der Charité (DHZC) – Medical Heart Center of Charité and German Heart Institute Berlin, Berlin, Germany; 5 Charité – Universitätsmedizin Berlin, Corporate Member of Freie Universität Berlin and Humboldt-Universität zu Berlin, Berlin, Germany

With great interest we read the letter to the editor from Prof. Haas and colleagues regarding our recently published publication “Chronic high consumption of energy drinks and cardiovascular risk in adolescents—results of the EDKAR-study” [1]. In this rejoinder, we respond to the concerns raised by the commentators and endeavor to clarify any misinterpretations of our work.

- **Inclusion and exclusion criteria**
  The inclusion and exclusion criteria for study phase 2 of the cross-sectional EDKAR-study were clearly defined. According to the definition in the EDKAR-study, participants with chronic high energy drink (ED) consumption are defined as adolescents consuming EDs at least 4 days per week for at least one year and exceeding the limit of 3 mg of caffeine from EDs per kilogram of body weight (kg BW) per day. Participants in the control group were adolescents who had not consumed EDs in the last 12 months and who consumed not more than 80 mg of caffeine per week from other caffeinated beverages (coffee drinks, black/green tea, drinking chocolate, cola and mate soft drinks, iced tea, pre-workout boosters). To verify these inclusion and exclusion criteria, data from the online questionnaire of study phase 1 was used to invite the identified adolescents to study phase 2 (cardiological examination).
- **Differences in various lifestyle and risk factors**
  As positively emphasized by Haas and colleagues, and also shown by previous studies, we were able to identify expected differences in various lifestyle factors (e.g., smoking, alcohol consumption, sleep duration, etc.) between chronic high ED consumers and the control group.
  Due to the use of a comprehensive online questionnaire in study phase 1, we have a wide range of important data on lifestyle and risk factors for each participant in study phase 2, enabling us to link the cardiological data with the data from the online questionnaire (assessed in study phase 1). As demonstrated in the publication, the results of all cardiologically measured parameters were presented as a simple comparison (unadjusted, Model 1) between chronic high ED consumers and the control group, but also taking into account (adjusting for) other important confounders (Model 2 and Model 3). In the present study, the confounder structure was determined using a directed acyclic graph (DAG) to estimate the minimal sufficient adjustment sets of potential confounders for the total effect of ED consumption on cardiological parameters (Supplement Fig. 1) [1]. Thus, the impact of the mentioned differences in these confounders between both groups was taken into account in the statistical analysis with regard to the investigation of cardiological parameters between chronic high ED consumers and the control group.
- “**Loss-to-follow-up**”
  As demonstrated in the publication, the planning of the EDKAR-study was based on a power calculation (see at power calculation in Menzel et al. [2]). Briefly, based on various cardiological parameters, 50–100 chronic high ED consumers and 100–200 controls should participate in the cardiological examination in order to be able to reveal statistically significant results. To achieve this, it was calculated that approximately 5000 adolescents would need to participate in the “screening study” of study phase 1. The first study phase was completed with a total of 5100 Berlin school pupils. Cardiological parameters were determined for a total of 99 chronic high ED consumers and 160 controls. Two individuals had to be excluded from the statistical analysis retrospectively. As depicted, all targeted sample sizes were achieved across study phase 1 and 2.
  Not all invited adolescents responded to the invitation and were cardiologically examined, but this was already taken into account in the power calculation. As a note, the “satisfactory” response rate refers to the fact, that the study team had no possibility of directly contacting invited pupils and motivating them to take part in study phase 2, but still the requested participant number was achieved on study phase 2.
  Comprehensive data of study phase 1 allowed the investigation of a possible selection bias (see Supplementary Table 9) in order to rule out the possibility that the adolescents who underwent cardiological examinations differed systematically in important characteristics from the adolescents who did not undergo examinations in both groups. However, this could not be seen for chronic high ED consumers (only minor differences in the control group) and therefore it cannot be assumed that the cardiological parameters differ systematically.
- **Definition of doses**
  The EFSA considered children and adolescents separately [3]. Accordingly, the EFSA opinion proposed a daily caffeine intake of 3 mg/kg BW/day for children and adolescents of no concern [3]. Based on this the definition of chronic high ED consumption was derived for the EDKAR-study. Thus, all chronic high ED consumers examined in the EDKAR-study had consumed EDs at least 4 days per week for at least one year and exceeded the limit of 3 mg caffeine from EDs/kg BW/day. The EFSA did not derive a recommended dose of 400 mg or 600 mg of caffeine for children and adolescents. We are pleased to provide further information in Table 1 on the ED consumption of chronic high ED consumers from study phase 2.

**Table 1:** Consumption of EDs, caffeine intake from EDs and bodyweight-adjusted caffeine intake from EDs of chronic high ED consumers in the EDKAR-study (study phase 2)

	Chronic high ED consumers (*n* = 97) median (IQR)
Consumption of EDs (liters/day)	1.0 (0.75–1.25)
Caffeine intake from EDs (mg/day)	320 (240–400)
Bodyweight-adjusted caffeine intake from EDs (mg/kg BW/day)	4.53 (3.69–6.08)
- **Cardiovascular examination**
  The EDKAR-study was designed as a cross-sectional study (observational study) suitable for investigating potential lasting effects of chronic high ED consumption. As the EDKAR-study is an observational study, participants are expected to behave as they would in their normal everyday lives—also referred to the day of cardiological examination. Therefore, in line with the study design, it is correct that we have no information on whether or when the participants had already consumed EDs on the day of the cardiological examination. Nevertheless, this is not relevant to the research question, which investigates whether chronic high ED consumption (for at least one year) already shows potential lasting changes/differences in cardiovascular parameters compared to a control group. The EDKAR-study did not examine whether acute consumption leads to short-term changes in cardiovascular parameters. Of course, it would have been desirable to take multiple time points of a blood pressure measurement for an even more detailed analysis. In fact, the suggested 24 h blood pressure measurement would also have been of interest in the context of chronic high ED consumption. However, it is important to note that a 24 h blood pressure measurement would also have recorded the acute effects of ED consumption (if EDs were consumed during this period). To avoid this, chronic high ED consumers would have had to abstain from consuming EDs on the day of the cardiological examination in the EDKAR-study in order to ensure that only potential lasting effects on blood pressure were measured. The same applies to the proposed use of a 24 h ECG. Of course, other parameters like the suggested arterial stiffness and carotid intima-media thickness would also have been interesting, but were not planned as part of the present study.
- **Application of reference values**
  Based on the physical constitution of all EDKAR participants (weight and height) who underwent cardiological examinations and their age of 15–18 years (girls: weight (mean ± standard deviation): 58.2 kg  ± 10.6; height: 165.5 cm ±  6.8; boys: weight: 71.0 kg ± 13.8; height: 177.9 cm ± 7.9), and their proximity to adult values, it is reasonable to use the references for adults. The average body surface area (BSA) of the participants is roughly equivalent to that of adults. When calculating Z-scores using the Pediatric Endocrinology Data Collection System (PEDZ, DGKED e. V., version 2025) [4], 34 participants cannot be calculated due to their body surface area (calculation limit BSA = 2 m^2^).
- **Data interpretation**
  We could not help to note that the alternative interpretation of our data offered by Haas et al. [1] is misleading, as study phase 2 of the EDKAR-study does not distinguish between chronic high ED consumers and high ED consumers—there is only a differentiation of chronic high ED consumers (*n* = 97) and the control group (*n* = 160).
  In the interpretation offered by Haas and colleagues they compare unadjusted geometric mean of all chronic high ED consumers (*n* = 97) with the unadjusted median of the control group (*n* = 160) and with the unadjusted median of a subgroup of the 10% of chronic high ED consumers with the highest caffeine intake from EDs (≥ 8.64 mg caffeine EDs/kg BW/day) in relation to diastolic blood pressure, septum thickness, E-wave and A-wave.
  To present a more appropriate comparison of the proposed interpretation, we provide in Table 2 the fully adjusted model for the mentioned parameter, since the strong confounders have already been addressed. The adjusted geometric mean for the control group changed because the calculation of least squares means from multivariable adjusted analysis of covariance (ANCOVA) is dependent on the overall covariate distribution of the pooled sample, which changed upon the restriction on 10% of chronic high ED consumers with the highest caffeine intake from EDs.

**Table 2:** Investigation of adjusted differences in diastolic blood pressure, IVSd, E-wave and A-wave between participants with chronic high ED consumption (*n* = 97) compared to the control group (*n* = 160) and 10% of chronic high ED consumers (≥ 8.64 mg caffeine from EDs/kg BW/day) (*n* = 10) compared to the to the control group (*n* = 160)

Chronic high ED consumers compared to the control group					
		Chronic high ED consumers (*n* = 97)	Control group (*n* = 160)	Difference	
	*n*	Geometric mean (95%-CI)^1^		Δ	*P-value*
DBP [mmHg]	228	76.6 (72.3–81.2)	76.2 (71.5–81.1)	0.45	*0.79*
IVSd [mm]	226	9.26 (8.44–10.2)	9.01 (8.15–9.97)	*0.25*	*0.44*
E-wave [cm/s]	224	86.1 (78.6–94.2)	90.3 (81.8–99.7)	*− 4.28*	*0.16*
A-wave [cm/s]	224	52.4 (46.5–59.1)	53.1 (46.6–60.4)	*− 0.68*	*0.78*

10% of chronic high ED consumers compared to the control group					
		10% of chronic high ED consumers (*n* = 10)	Control group (*n* = 160)	Difference	
	*n*	Geometric mean (95%-CI)		Δ	*P-value*
DBP [mmHg]	154	79.4 (69.5–90.7)	76.3 (67.9–85.7)	3.15	*0.59*
IVSd [mm]	153	9.70 (7.79–12.1)	9.14 (7.54–11.1)	0.56	*0.64*
E-wave [cm/s]	152	80.2 (64.1-100.5)	98.1 (80.5-119.4)	− 17.8	*0.12*
A-wave [cm/s]	152	40.8 (30.5–54.7)	54.6 (42.3–70.6)	− 13.8	*0.08*

^1^Except of Δ, already published in [1]; adjusted for age, sex, physical activity, smoking status, smoking cannabis, alcohol consumption and school type; varying numbers of study participants in the cardiological parameters are caused by missing parameter measurements and/or missing information of included confounders

Even though more pronounced differences/change (not statistically significant) might be seen in the subgroup of the 10% highest chronic ED consumers with very high consumption of EDs (≥ 8.64 mg caffeine EDs/kg BW/day) vs. control group, compared to all chronic high ED consumers vs. control group, caution is needed in the interpretation. The respective trend interpretation is based on one (the smallest) of the many subgroup analyses. Due to the low number of participants (*n* = 10) in the referring subgroup analysis, the results are less reliable than the main analyses and the probability of false conclusions is increased. Consequently, an overinterpretation of these subgroup results cannot be ruled out in the interpretation by Haas and colleagues. An explicit attention was pointed to the partly low number of participants in the subgroup analyses in the publication, as this limits the reliable interpretation of the data. Further research is needed. Taken together, in our view the proposed “subtle” trends, especially proposing this as main results of the study, are not scientifically reasonable, since the respective trend interpretation refers only to one subgroup analysis and does so with a small sample size/ low power (*n* = 10) without consideration of all other analyses.

## Concluding remarks

The concerns raised in the comments do not necessitate altering our original inferences and conclusions. Given that the EKDAR-study is the only (cross-sectional) study so far that investigates the chronic high ED consumption (not acute ED consumption) in relation to cardiovascular parameters, we hope that future research on the associations between chronic high ED intake and cardiovascular risk in adolescents will provide additional evidence in this area of research.
